# Recurrent Airway Swelling in a Patient With Lymphoproliferative Disorder: A Diagnostic Challenge Between Anaphylaxis and Acquired Angioedema

**DOI:** 10.7759/cureus.96070

**Published:** 2025-11-04

**Authors:** Mahmudul Hasan Nahid, Bilal Khan, Reema Munshi

**Affiliations:** 1 Acute Internal Medicine, University Hospitals of Leicester, Leicester, GBR; 2 Internal Medicine, Shaheed Ziaur Rahman Medical College, Bogura, BGD; 3 Emergency Department, Lancaster Royal Infirmary, Lancaster, GBR; 4 Internal Medicine, University Hospitals of Leicester, Leicester, GBR

**Keywords:** acquired angioedema, bradykinin mediated angioedema, c1 esterase inhibitor deficiency, icatibant therapy, lymphoproliferative disorder, refractory anaphylaxis

## Abstract

Distinguishing between anaphylaxis and bradykinin-mediated angioedema can be difficult in the acute setting, particularly when airway compromise dominates the presentation. Recognition is crucial, as management strategies differ substantially. An 84-year-old woman presented to the Emergency Department in Preston with rapidly progressive swelling of the lips, tongue, and neck, accompanied by dysphagia and a hoarse voice. She had self-administered multiple doses of an adrenaline autoinjector before arrival. On examination, she was hemodynamically stable and afebrile, with marked tongue and floor-of-mouth edema but no urticaria or pruritus. Considering refractory anaphylaxis, she received intravenous dexamethasone, an adrenaline nebulizer, and an adrenaline infusion. ENT and ICU teams were involved; flexible nasendoscopy confirmed edema of the floor of the mouth and arytenoids, with mild epiglottic involvement but an adequate glottic opening. This episode followed several similar admissions since 2022, each previously treated as anaphylaxis with limited response to adrenaline. Further investigation revealed normal mast-cell tryptase (6.1 µg/L) and persistently low C4 (<0.03 g/L) with normal C3 (1.29 g/L), findings consistent with C1-esterase inhibitor (C1-INH) deficiency. Given her history of CD5-negative low-grade B-cell lymphoproliferative disorder, a diagnosis of acquired angioedema was made following multidisciplinary review, and treatment with icatibant was initiated. This case highlights the importance of considering bradykinin-mediated angioedema in patients with recurrent “refractory anaphylaxis.” The absence of urticaria, normal tryptase, and low complement levels should prompt evaluation for C1-INH deficiency, allowing timely, targeted management and improved outcomes.

## Introduction

Angioedema is a localized, self-limiting swelling of subcutaneous or submucosal tissue caused by transient increases in vascular permeability. It may occur through histamine-mediated mast-cell activation, as in allergic anaphylaxis, or through bradykinin excess resulting from C1-esterase inhibitor (C1-INH) deficiency [1]. The acquired form (acquired angioedema due to C1-esterase inhibitor (AAE-C1-INH) deficiency) arises from immune-mediated consumption or inhibition of the inhibitor, most commonly secondary to B-cell lymphoproliferative or autoimmune disease.

AAE-C1-INH is rare, with an estimated prevalence of about one in 100000 people and accounting for fewer than 10% of all C1-INH deficiencies [2]. Clinically, it may present with recurrent non-pitting edema of the face, tongue, or airway, but lacks urticaria or pruritus. Its airway involvement and rapid onset may resemble anaphylaxis; patients are frequently misdiagnosed and treated repeatedly with adrenaline, corticosteroids, and antihistamines, which provide little benefit [3].

Here we present the case of an elderly woman with recurrent episodes of adrenaline-resistant airway swelling initially labelled as refractory anaphylaxis, later diagnosed as acquired C1-INH deficiency secondary to a low-grade B-cell lymphoproliferative disorder. The case highlights how bradykinin-mediated angioedema can mimic severe allergic reactions and underscores the importance of complement testing and multidisciplinary review in patients with atypical or treatment-resistant anaphylaxis, as bradykinin-mediated angioedema does not respond to adrenaline, antihistamines, or corticosteroids. These treatments are ineffective and may delay appropriate care.

## Case presentation

**Figure 1:**
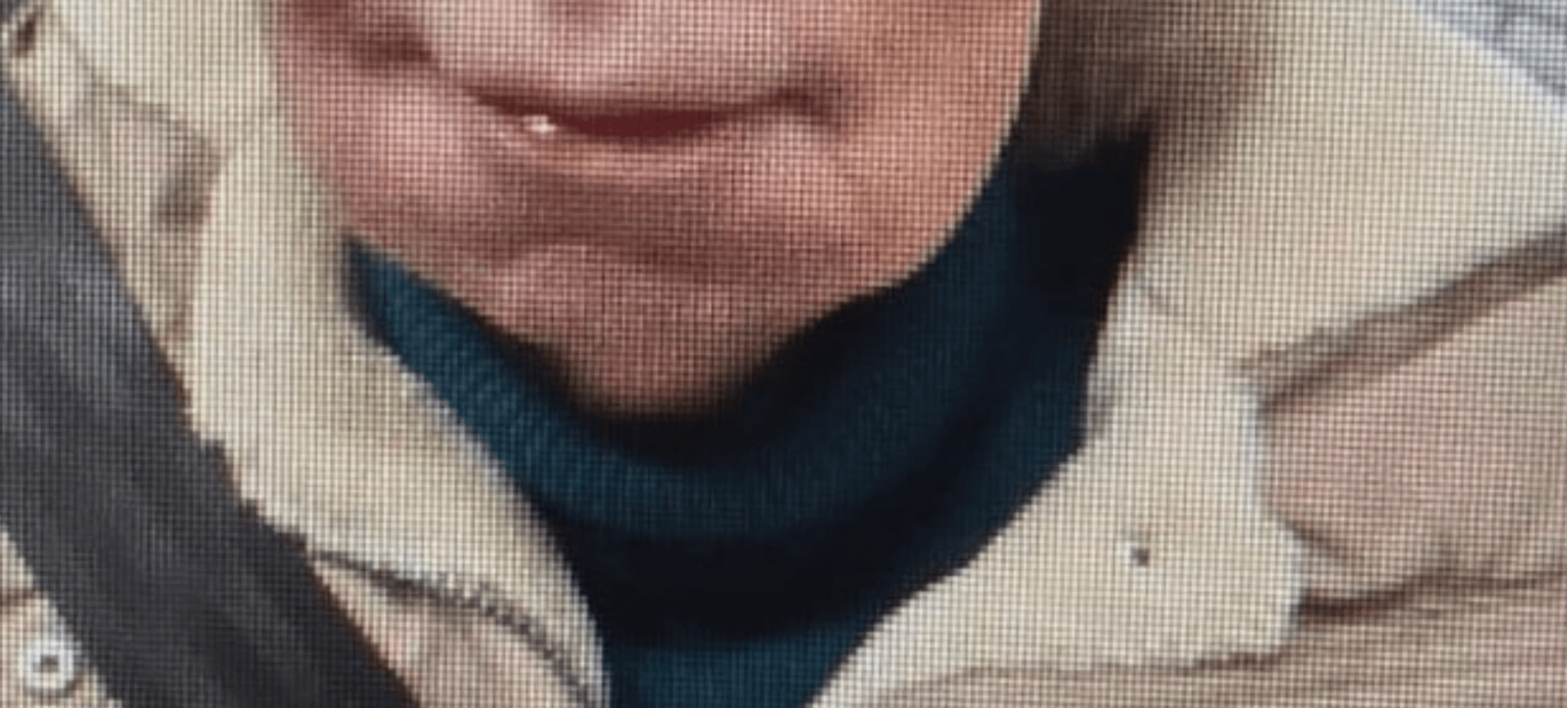
Clinical photograph at presentation showing swelling of the floor of the mouth, consistent with acute airway edema in acquired C1-INH deficiency. The tongue is partially obscured due to positioning C1-INH: C1-esterase inhibitor

An 84-year-old woman presented to the Emergency Department in Lancaster with rapidly progressive swelling of the lips, tongue, and neck, accompanied by dysphagia and a hoarse voice. She had administered multiple doses of adrenaline autoinjector (reported five) before arrival. On examination, she was alert, saturating well on room air, and hemodynamically stable, but had marked swelling of the tongue and floor of the mouth (Figure 1). No urticaria or pruritus was noted. The patient denied abdominal pain, nausea, or any gastrointestinal symptoms during this or prior episodes, and there were no clinical findings to suggest gastrointestinal involvement. She received intravenous dexamethasone, an adrenaline nebulizer, and was commenced on an adrenaline infusion. ENT and ICU were urgently involved; flexible nasendoscopy demonstrated floor-of-mouth and arytenoid edema with mild epiglottic swelling but an adequate glottic opening. Awake fiber-optic intubation was considered if she deteriorated. Over the following hours, her voice improved, and the swelling began to resolve.

This was the latest in a series of admissions since 2022 for similar airway swelling, each previously managed as anaphylaxis. On those occasions, she had self-administered adrenaline prior to arrival and was treated with repeated intramuscular and nebulized adrenaline, intravenous corticosteroids, and antihistamines. Despite this, her symptoms often settled only slowly and were described as “Refractory anaphylaxis.” Across all episodes, she remained hemodynamically stable, and urticaria was never documented. The absence of typical allergic features and repeated poor response to adrenaline prompted consideration of alternative diagnoses.

**Figure 2:**
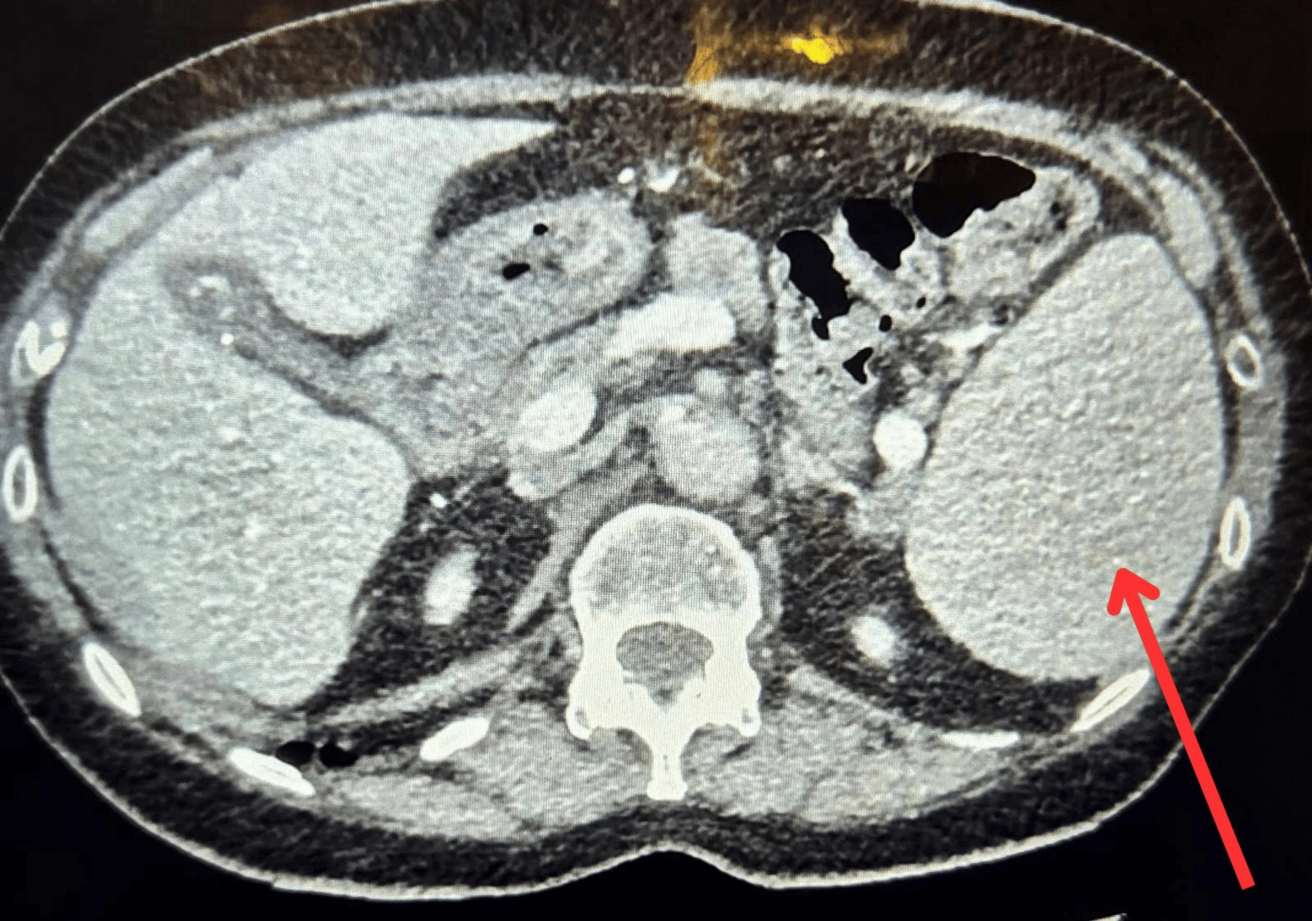
CT scan of the thorax, abdomen, and pelvis shows splenomegaly

Her background included a CD5-negative low-grade B-cell lymphoproliferative disorder, diagnosed on bone-marrow biopsy in 2018, which was managed conservatively under hematology follow-up, with no evidence of systemic lymphadenopathy or disease progression on serial imaging. Serial imaging demonstrated progressive splenomegaly (16 cm in 2018, 22 cm in 2022/23, and regressing to 18.4 cm by May 2025) as shown in Figure 2. A CT scan in May 2025 also revealed a progressive spiculated lesion in the left lower lobe (2.1-2.7 cm), concerning for either primary lung malignancy or lymphomatous involvement. No cervical or thoracic lymphadenopathy was seen. The patient’s regular medications were reviewed, and she was not taking any ACE inhibitors, angiotensin receptor blockers, or non-steroidal anti-inflammatory drugs (NSAIDs), which were therefore excluded as potential contributors to her angioedema.

The case was reviewed at the regional lung cancer multidisciplinary team (MDT), but the patient declined an invasive biopsy, and a plan for surveillance imaging was agreed upon. During this admission in February 2025, further investigations were pursued. Serum mast-cell tryptase was 6.1 µg/L (within the normal range). Complement studies showed normal C3 (1.29 g/L) with persistently low C4 (<0.03 g/L), findings strongly suggestive of C1-INH deficiency. Quantitative and functional C1-INH assays were subsequently completed by the regional Immunology Department during outpatient follow-up, confirming acquired C1-INH deficiency. The exact values were not accessible at the time of writing. However, routine blood tests were otherwise reassuring: the full blood count was normal, renal function stable (urea 6.4 mmol/L; creatinine 94 µmol/L), with only mild hyponatremia (131 mmol/L) and hypochloremia (94 mmol/L). A full panel of investigation results is shown in Table 1.

**Table 1:** Patient's investigation results with respective reference ranges MCV: mean cell volume

Investigation name	Value	Unit	Reference range
Hemoglobin	134	g/L	115-165
WBC count	5.9	x10^9^/L	4.0-10.0
Platelet count	203	x10^9^/L	150-400
Hematocrit	39.9	%	36-40
Red blood cell count	4.2	x10^12^/L	3.80-5.80
MCV	95.1	fL	77-100
Mean cell hemoglobin	31.8	pg	27-32
Neutrophil count	2.5	x10^9^/L	2.0-7.0
Monocyte count	0.3	x10^9^/L	0.2-1.0
Basophils	0	x10^9^/L	0.00-0.10
Lymphocyte count	2.6	x10^9^/L	1.0-3.0
Eosinophil count	0.5	x10^9^/L	0.02-0.54
Sodium	131	mmol/L	133-146
Potassium	4	mmol/L	3.5-5.3
Chloride	94	mmol/L	95-108
Urea	6.7	mmol/L	2.5-7.8
Creatinine	94	mmol/L	49-90
Mast-cell tryptase level	6.1	ug/L	2.0-14.0
Complement C3 level	1.29	g/L	0.75-1.65
Complement C4 level	<0.03	g/L	0.14-0.54

In light of the recurrent airway episodes, poor response to adrenaline, normal tryptase, and persistently low C4 in the setting of lymphoproliferative disease, the working diagnosis was revised to AAE-C1-INH deficiency.

The case was discussed with multiple departments, including hematology, respiratory medicine, emergency care, and the Immunology Department. She was discharged once stable, with icatibant prescribed for self-administration, clear escalation instructions, and a prioritized outpatient immunology appointment, in addition to ongoing hematology follow-up, which later on finalized the diagnosis. At her most recent review in July 2025, she remained clinically stable, with preserved counts (Hb 125 g/L, WCC 4.9×10⁹/L, platelets 145×10⁹/L, LDH 205 U/L) and a stable weight (69.6 kg). Splenomegaly was not thought to be driving her symptoms. The pulmonary lesion is being monitored, with a repeat CT chest scheduled in six months. The lesion was considered radiologically stable and not thought to contribute to her episodes of angioedema.

## Discussion

AAE-C1-INH deficiency is an uncommon but well-recognized clinical entity, with an estimated prevalence of approximately one in 100000 people and accounting for fewer than 10% of all C1-INH deficiencies [1,2]. Although rare, it is frequently misdiagnosed; in one multicenter review, almost one-third of patients were initially treated for recurrent allergic or “idiopathic” angioedema before the underlying mechanism was identified [3]. This case shows how a patient with multiple documented allergies and dramatic airway swelling can cycle through repeated episodes of refractory anaphylaxis before the true mechanism is recognized. Each episode was managed appropriately according to national anaphylaxis protocols. Yet, the absence of a clear allergen exposure, lack of urticaria, preserved hemodynamics, and poor response to adrenaline gradually challenged the initial assumption. The decisive step was to reinterpret these “refractory” episodes in light of her known low-grade B-cell lymphoproliferative disorder, a context in which acquired C1-INH deficiency becomes pathophysiologically plausible and clinically significant.

The repeated episodes in this patient were initially managed as anaphylaxis according to national emergency protocols [4]. However, several consistent clinical findings gradually challenged that assumption. Each episode presented with prominent oropharyngeal edema but without hypotension, urticaria, or bronchospasm, features that occur in more than 80% of confirmed mast-cell-mediated anaphylaxis [5]. During the index admission, serum mast-cell tryptase obtained within hours of onset measured 6.1 µg/L. A prospective study analyzed 102 patients with suspected anaphylaxis and demonstrated that a peak tryptase value exceeding 11.4 µg/L strongly supported the diagnosis, whereas normal results were not compatible with systemic mast-cell activation [6]. In this patient, a normal tryptase level therefore argued against an IgE-mediated process. Complement studies then revealed persistently low C4 with normal C3, a biochemical pattern regarded as characteristic of C1-INH deficiency and recommended by the World Allergy Organization/European Academy of Allergy and Clinical Immunology (WAO/EAACI) guideline as the initial screening clue before measuring C1-INH concentration and function [7,8].

Interpreting these “refractory” episodes in the context of her known low-grade B-cell lymphoproliferative disorder provided the critical diagnostic link. A review paper demonstrated that patients with such lymphoid proliferations may develop circulating anti-C1-INH autoantibodies or increased consumption of the inhibitor, producing functional deficiency and recurrent bradykinin-mediated angioedema [9]. Because bradykinin increases vascular permeability through endothelial B2-receptor activation rather than mast-cell degranulation and is associated with C1-INH-deficient angioedema, the resulting edema is inherently unresponsive to adrenaline, corticosteroids, or antihistamines [10]. Recognizing this mechanism explained the poor response to prior treatment and marked the turning point in establishing the correct diagnosis.

In IgE-mediated anaphylaxis, cross-linking of allergen-specific IgE on mast cells and basophils causes rapid degranulation and release of histamine, platelet-activating factor, tryptase, and leukotrienes. These mediators produce vasodilation, increased vascular permeability, and smooth-muscle contraction, leading to the characteristic combination of urticaria, angioedema, bronchospasm, and hypotension [11]. Adrenaline remains the definitive treatment because its α-adrenergic effects reverse vasodilation and vascular leak, while β₂-receptor stimulation relaxes airway smooth muscle and suppresses further mediator release from mast cells [5]. Refractory anaphylaxis is defined by persistence of life-threatening symptoms despite at least two appropriately timed doses of intramuscular adrenaline and is uncommon. Reports from specialist centers estimate its frequency at less than 1% of all anaphylaxis presentations, most often linked to delayed adrenaline use, ongoing allergen exposure, or β-blocker therapy that blunts adrenergic response [12,13]. In this case, none of these factors were present: adrenaline was given promptly, no identifiable trigger was found, and cardiovascular stability was preserved throughout. These inconsistencies, together with the absence of urticaria and a normal tryptase concentration, made true refractory anaphylaxis unlikely and pointed instead to a non-mast-cell pathway for edema formation.

Acquired C1-INH deficiency reflects immune-mediated inactivation or consumption of the inhibitor rather than reduced synthesis. In a focused review of AAE associated with B-cell lymphoproliferative disorders, Castelli et al. described neutralizing anti-C1-INH autoantibodies and immune-complex-driven consumption as the principal mechanisms lowering antigenic and functional C1-INH, with angioedema activity often tracking the hematologic process [9]. Experimental work demonstrated that patient IgG can bind to and neutralize C1-INH, thereby removing control over C1r/C1s and kallikrein and permitting continuous activation of the classical complement and contact systems [10]. The resulting bradykinin excess, rather than histamine, drives endothelial B2-receptor-mediated permeability, explaining the absence of response to adrenaline, corticosteroids, and antihistamines in our patient [10].

Once a bradykinin pathway was identified, acute treatment appropriately shifted to bradykinin antagonism or C1-INH replacement, which acts directly on the dysregulated cascade rather than mast-cell mediators. Icatibant blocks bradykinin at the B₂ receptor. In the New England Journal of Medicine (NEJM) program For Angioedema Subcutaneous Treatment (FAST)-2 (active-comparator trial), it shortened time to symptom relief (median 2.0 h vs 12.0 h with tranexamic acid), while FAST-1 (placebo-controlled) did not meet its primary endpoint largely due to early rescue medication, though secondary measures favored icatibant [14]. For AAE, an open-label multicenter series documented successful on-demand icatibant use during acute attacks [15]. Plasma-derived C1-INH (pd-C1-INH) restores inhibitor activity and directly reins in kallikrein and the classical complement pathway. Randomized controlled trials in hereditary C1-INH deficiency demonstrated faster clinical resolution versus control and underpin extrapolation for AAE acute attacks where available [7,8]. Ecallantide (a plasma kallikrein inhibitor) has also shown benefit in placebo-controlled trials for acute hereditary angioedema (HAE), offering another mechanism-level option when accessible [16]. Current international guidance therefore recommends icatibant or pd-C1-INH as first-line agents for acute attacks of C1-INH deficiency, reserving corticosteroids and antihistamines for histaminergic disease [8]. For disease control, management of the underlying B-cell clone is pivotal in AAE: case series report rituximab-based regimens achieving biochemical normalization and sustained remission of angioedema where autoantibody-mediated C1-INH loss is suspected [9]. In our patient, clinical stability of the lymphoproliferative process paralleled symptom improvement, consistent with this mechanism-directed approach. For long-term prevention in C1-INH deficiency, kallikrein inhibition (e.g., Lanadelumab; oral Berotralstat) and regular pd-C1-INH reduce attack rates in hereditary disease; in AAE, prophylaxis is individualized and often secondary to controlling the hematologic driver [8,17].

Published series of acquired C1-INH deficiency consistently describe cutaneous and abdominal swelling as the dominant manifestations, with airway compromise reported far less frequently [9]. Unlike those cohorts, this case presented almost exclusively with recurrent laryngeal and oropharyngeal edema that was repeatedly treated as anaphylaxis despite hemodynamic stability and normal tryptase. The presentation, therefore, broadens the recognized clinical spectrum of AAE-C1-INH and underscores how bradykinin-mediated airway involvement can mimic refractory anaphylaxis even when classical allergic features are absent.

## Conclusions

This case reinforces that adrenaline-resistant airway swelling without urticaria or raised tryptase should prompt evaluation for bradykinin-mediated angioedema. Early measurement of complement components (C4 and C1-INH level and function) can prevent diagnostic delay and avoid repeated ineffective therapy. Recognition of the link between lymphoproliferative disease and acquired C1-INH deficiency allows timely initiation of targeted treatment and multidisciplinary follow-up, improving both acute outcomes and long-term disease control.
